# Appropriateness of acute admissions and last in-patient day for patients with long term neurological conditions

**DOI:** 10.1186/1472-6963-9-40

**Published:** 2009-02-27

**Authors:** Christina L Hammond, Margaret F Phillips, Lorraine L Pinnington, Benedict J Pearson, Apostolos Fakis

**Affiliations:** 1 Rehabilitation Research and Education Group, School of Community Health Sciences, University of Nottingham, UK; 2 Rehabilitation Research and Education Group, School of Graduate Entry Medicine & Health, University of Nottingham, UK; 3 Department of Assessment and Diagnostics, Derby Hospitals NHS Foundation Trust, Derby, UK; 4 Research and Development Department, Derby Hospitals NHS Foundation Trust, Derby, UK

## Abstract

**Background:**

To examine the appropriateness of admissions and in-patient stay for patients with long term neurological conditions (LTNCs). To identify variables predictive of appropriateness and explore management alternatives.

**Methods:**

Adults admitted as acute patients to Derby Hospitals NHS Foundation Trust (England). Data were collected prospectively and examined by a multi-disciplinary expert panel to determine the appropriateness of admission and length of stay (LoS). Management alternatives were discussed.

**Results:**

A total of 119 participants were recruited. 32 admissions were inappropriate and 83 were for an inappropriate duration. Whether a participant lived in their own home was predictive of an inappropriate admission. The number of LTNCs, number of presenting complaints and whether the participant lived alone in their own home were predictive of an inappropriate LoS. For admissions judged to be inappropriate, the panel suggested management alternatives.

**Conclusion:**

Patients with LTNCs are being admitted to hospital when other services, e.g. ambulatory care, are available which could meet their needs. Inefficiencies in hospital procedures, such as discharge planning and patient transfers, continue to exist. Recognition of the need to plan for discharge at admission and to ensure in-patient services are provided in a timely manner may contribute towards improved efficiency.

## Background

People with long term neurological conditions (LTNCs) sometimes have acute episodes of illness that may necessitate hospital admission. The need for hospital admission and continued hospital stay should be dependent on the type of assessment, observation and intervention required for the presenting condition. However, studies from the UK and elsewhere have shown that a number of patients are admitted to hospital, and/or remain in hospital when their needs (for assessment, observation and/or intervention) can be met more effectively elsewhere [1-6]. Although the proportions of inappropriate admissions 6–31% [1,3,5-7] and of length of stay 14–66% [3,5,6] vary widely, many of these discrepancies can be attributed to methodological differences between studies. Also, the discrepancies may relate to variations in provision of care from country to country, and the age of the study, with a number of studies cited pre-dating key policy developments, for example the commitments outlined in the NHS plan [8]. Although McDonagh, noted when reviewing evidence for a systematic review, that it can be difficult to draw conclusions reliably from existing data, she suggests that the percentage of inappropriate acute hospital bed use for adults, worldwide, lies somewhere between 15% and 50% [9]. The importance of this topic has been reflected in the recent policy agenda. The National Service Framework (NSF) for Long Term (neurological) Conditions, for example, outlines quality requirements for the care and treatment of patients with LTNCs admitted to hospital, and aims to contribute towards the Public Service Agreement target of 'reducing emergency bed days by 5% by 2008 through improved care in primary and community settings for people with long-term conditions' [10].

The age, health and functional status of the patient and the speciality of care admitted to have all been shown to be associated with inappropriate admissions [3,11]. Living alone, absence of family support and the requirement for home nursing following discharge or entry to an institutional care facility, contribute to an increased likelihood of adult and older patients remaining in hospital unnecessarily [12-16]. It is not clear what leads to patients being admitted to hospital inappropriately but it may be partly attributable to wider problems such as the availability of respite care facilities and/or long term care institutions being limited [3,6,8,17-19].

A systematic review that examined delayed discharge showed that: poor communication between hospital and community services; lack of assessment and discharge planning; inadequate notice of discharge; inadequate consultation with patients and their carers; over-reliance on informal support and lack of (or slow) statutory service provision; and inattention to the special needs of vulnerable groups are problems that characterise delayed hospital discharge [20].

A limited number of studies have examined acute neurological conditions, such as a stroke, and have shown a proportion of the admission is medically inappropriate in 21–50% of patients and 36% of all in-patient days are inappropriate [21-24]. In one UK study, it was revealed that 88% of adult patients with a delayed discharge had a primary neurological diagnosis [18]. Awaiting provision of social care, home adaptations and transfer to another care facility are all associated with an inappropriate LoS amongst this patient population [18,23-25]. Similarly, a study of patients with brain injury revealed that failure to find a suitable placement and failure to obtain funding for post discharge support were common causes of delayed discharges [26].

Any person admitted to hospital is placed at risk of hospital acquired infection (often multi-resistant organisms), increased immobility, increased dependency and social isolation [27-30]. For those with a LTNC, acute hospitals may not be the most appropriate setting in which to address the needs that commonly arise from a LTNC (such as bowel management and chair or bed positioning). In order to ensure patients with LTNCs are treated in an appropriate environment, we need to know whether patients are being admitted when they do not need to be or if they are staying in hospital longer than is necessary. We also need to understand what factors are associated with inappropriate admissions and LoS, and which non-acute services could meet the needs of this population.

## Methods

### Study aims

The study aimed to identify

1. the prevalence of inappropriate admissions and lengths of in-patient stay amongst patients with LTNCs

2. the form(s) of care that would be the most suitable alternative when an admission was deemed inappropriate

3. the actions that would have facilitated an appropriate length of stay when the length of stay was deemed inappropriate

4. variables predictive of inappropriateness

### Participants and Setting

Patients were recruited from the Medical Assessment Unit (MAU) of Derby Hospitals NHS Foundation Trust. The Trust comprises two acute hospitals which have 56 assessment and 352 general medical beds. Collectively, the hospitals serve a population of approximately 600,000 people.

### Inclusion criteria

Patients meeting the following criteria were eligible for inclusion in the study:

1. Admission to a medical ward in an acute trust

2. Patients aged 18 years or above

3. A diagnosis, made by a physician, of one or more LTNC present for 12 months or more

4. Acute stroke (if the event had resulted in lasting impairment, i.e. 12 months or more)

### Procedure

Participants were recruited from two acute hospital sites in Derby (i.e. Derbyshire Royal Infirmary/Derby City General Hospital) between June 2006 and January 2007. The health records of patients admitted to the MAU were reviewed to determine those who were eligible for recruitment (i.e. were documented as having a LTNC in their health records). Those who were eligible for recruitment were approached (chronologically) within one to two days of being admitted and were provided with a copy of the relevant study documentation (an information sheet and consent form). A clinician involved in the care of the patient, i.e. a nurse or physician, provided their professional opinion as to whether or not they felt the patient was able to provide informed consent. In line with the Mental Capacity Act 2005 permission was sought to recruit patients who were unable to provide informed consent. For those patients who were unable to give informed consent, a member of their family or a friend was approached to provide assent on the patient's behalf. Where there were no family members or friends visiting, ethical permission was given to collect basic information from the patient's health records. Ethical approval was obtained from the Derbyshire NHS Research Ethics Committee (06/q2401/60).

### Data collection

A researcher collected data from the health records which included: demographics, use of community health services, medical history and the admitting problem, in-patient management and outcomes of admission. In addition, structured interviews were carried out with patients at the bedside and data collected by this method included: use of community health services, the availability of formal or informal care, the circumstances surrounding the admission, level of functional ability, dependence and cognitive status. Information regarding the participant's functional abilities, level of dependence and cognitive status were assessed using three tools, namely the Guy's Neurological Disability Scale (GDNS), the Functional Independence Measure (FIM), and the Mini Mental State Examination (MMSE) [31-33].

### Assessment of appropriateness

The appropriateness of the admission and LoS was assessed by an expert panel. In contrast to 'appropriateness tools' (such as the Appropriateness Evaluation Protocol [34]) the 'expert panel method' allowed a specific assessment of the circumstances surrounding each admission and LoS to be considered in depth and to list the service areas (including neurological services) where alternative management options existed.

The panel consisted of a neurological rehabilitation physician, an acute care physician and a general practitioner. In order to standardise the process and maintain parity between cases, panel members adhered to a working definition when determining whether or not the admission/length of stay was appropriate.

'Admissions and lengths of stay are deemed appropriate when the level of care required by the patient can only be provided in a hospital e.g. they require access to specialist equipment, treatment administration such as intravenous antibiotics, or urgent specialist input.'

Each panel member was presented with the data collected (background information including demographic information, medical history etc, the in-patient case notes and a summary of the GDNS, FIM and MMSE assessment outcomes). Expert panel members commented on the medical necessity of the admission/length of stay. When an admission was judged to be inappropriate the length of stay was also classified as inappropriate. Therefore, the lengths of stay of only those who had been admitted appropriately were taken forward for analysis. Members examined the in-patient case notes of each participant and identified if the discharges were premature or delayed. Due to time constraints, the appropriateness of each in-patient day could not be assessed during the panel meetings. Therefore, the panel examined the in-patient case notes relating to the last three to four days of admission to determine the appropriateness of continued admission (and to identify causes of delay where relevant). As the panel did not examine the whole length of stay (for patients whose stay was greater than three/four days) it was not possible to determine the number of days in which a delay occurred. The expert panel therefore assessed the appropriateness of the final day spent in hospital.

To minimise bias, panel members were asked to consider the appropriateness of the admission independently and without examining in-patient case notes. They were also asked to reach their decision prior to the panel meeting and to provide the researcher with their preliminary decision. During panel meetings, members discussed their preliminary findings and provided their final decision regarding the appropriateness of each admission and LoS. The decision of the majority was taken forward as the final panel decision.

### Inter and intra rater reliability

The inter-rater reliability of decisions made independently and prior to the panel meetings showed that there was moderate agreement between raters (range: 0.419–0.444) when judging the appropriateness of admissions, as measured by the Kappa statistic [35]. The intra-rater reliability, referring to the agreement between individual's baseline decisions (those made before the expert panel) and the overall panel decision, showed there was moderate agreement with consensus being achieved in 79% to 90% of cases. When assessing the appropriateness of lengths of stay, there was moderate agreement between raters (inter-rater reliability) between their baseline decisions (range: 0.467–0.556). The intra-rater reliability was moderate to good with consensus being achieved in 76% to 84% of cases.

### Identifying alternative forms of care

Once the appropriateness of the acute admission and length of stay had been assessed, alternative forms of care were examined. In addition to the three physicians, a community matron, a social services manager, a neurological rehabilitation nurse specialist, an occupational therapist and a physiotherapist worked together to suggest alternative services or management strategies for the cases discussed. The inclusion of these additional members ensured that clinicians central to the admission, management and discharge of patients with LTNCs were represented and those selected had relevant clinical experience and current knowledge of the problems prevalent amongst patients with LTNCs. Involvement of a multi-disciplinary group ensured that alternative management strategies/services were examined from a variety of perspectives, thereby overcoming some of the limitations in knowledge that single clinicians may encounter (e.g. unfamiliarity with primary and secondary services).

### Estimation of sample size

Data from acute stroke studies were used to determine the sample size [22,24]. Based on this, inappropriate LoS was estimated to occur in approximately 35% of admissions. In order to construct a 95% confidence interval for this proportion with 5% precision (that is, + 5%), a sample of 350 persons was required. However, in this study the percentage of inappropriate LoS was 72% (83 out of 115 participants). Therefore, the 95% confidence interval that we estimated had a precision of 8%.

### Analysis

Descriptive statistics, [means (measure of spread – Standard Deviation), medians (measure of spread – Interquartile Range) and proportions] were used to describe participants and the outcomes of expert panel meetings. A Chi-Squared Test or Fisher Exact Test was used, where appropriate, to determine whether a difference existed in the proportion of participants admitted appropriately/inappropriately or whose last in-patient day was appropriate/inappropriate for specific categorical variables. The distribution of variables was examined for normality using the Kolmogorov-Smirnov test and histograms. The Mann Whitney U test was used to examine continuous variables between the two cohorts (appropriate/inappropriate admission or length of stay). Logistic regression was used to model variables predictive of an inappropriate admission and length of stay. Variables that were associated significantly with an inappropriate admission/length of stay after the Univariate analysis were entered into the logistic regression model separately for estimating the unadjusted Odds Ratios (OR) and 95% confidence intervals (CI). All the variables that were associated significantly with an inappropriate admission/length of stay were entered into one logistic regression model for estimating the adjusted OR and 95% CI. Using a Forward Stepwise Likelihood ratio selection method, variables were tested for significance. Entry of variables to the model was determined using the Log-Likelihood ratio test, with p < 0.05. The Hosmer-Lemeshow test was used for examining the goodness of fit for the model. Variables were deemed significant if the p-value was less than 0.05.

## Results

### Participant characteristics

A total of 298 patients met the eligibility criteria and of these 119 were recruited. The most common reason for failure to recruit eligible patients was that the patient was discharged before consent/assent could be obtained (n = 120, 67%), followed by the patient declining (n = 33, 18%). The calculated sample size was therefore not achieved. The confidence intervals of the proportion of patients who were admitted inappropriately/had an inappropriate length of in-patient stay and of the variables predictive of appropriateness are therefore less precise. However, aims one to three are unaffected.

Participants were recruited over an eight month period (June 06–January 07). Half of the sample were male, the majority were white (97%), living in their own home (79%) and had a median age of 73 years (58–82). The majority of participants had one LTNC (80%, n = 95), however, approximately one fifth of the participants had multiple LTNCs (20%, n = 24). The most frequent LTNC was a stroke (n = 56, 39%), followed by epilepsy (n = 31, 21%) Parkinson's disease (PD) (n = 18, 12%), dementia (n = 13, 9%), multiple sclerosis (n = 12, 8%), Alzheimer's disease (n = 4, 3%), sub-arachnoid haemorrhage (n = 4, 3%), peripheral neuropathy (n = 2, 1%). Chest pain (n = 14, 12%), shortness of breath (n = 11, 9%), collapse (n = 10, 8%) and a fall (n = 10, 8%) were the most common presenting complaints. Additional complaints included epileptic fit (n = 7, 6%), reduced mobility (n = 5, 4%), diarrhoea (n = 5, 4%) and participants being described as 'off their legs' (n = 5, 4%). In total, there were 39 different presenting complaints (see Table 1 for further details).

**Table 1:** Presenting complaints

**Presenting condition/complaint**	**Frequency**	**Percentage**
Chest pain	14	11.8
Shortness of breath	11	9.2
Collapse	10	8.4
Fall	10	8.4
Epileptic fit	7	5.9
Reduced mobility	5	4.2
Diarrhoea	5	4.2
Off their legs	5	4.2
Unwell	5	4.2
Left sided weakness	5	4.2
Raised blood sugar levels	4	3.4
Confusion	3	2.5
Vomiting	3	2.5
Overdose	2	1.7
Chest infection	2	1.7
Urinary tract infection	2	1.7
Dysphasia	2	1.7
Headache	2	1.7
Abdominal pain	2	1.7
Lethargy	2	1.7
Other	18	15.1

**Total**	**119**	**100%**

### Inappropriate admissions

The expert panel was not able to determine the appropriateness of three admissions and these were therefore excluded from the analysis. 32 out of 116 admissions were judged inappropriate (28%, 95% CI: 20.2–36.3). The most frequent reasons (sometimes more than one per participant) for an admission to be deemed inappropriate were that the participant's concern could have been managed by primary care services (40%); required attendance rather than admission to hospital (40%); required admission to a non-acute facility (13%); could have been managed by an outpatient service (8%). Of the alternative forms of care suggested by the expert panel, 15 cases (38%) were thought to be suitable for assessment or monitoring in a clinical decision unit only (without acute admission), 11 cases (28%) for health or social care at home, seven cases (15%) for respite or palliative care in a nursing home and four cases (13%) for a non acute hospital admission.

### Inappropriate length of stay

The LoS of those participants who were found to have been admitted to hospital appropriately (n = 81) were analysed. The expert panel was unable to provide a definitive answer regarding the appropriateness of three in-patient stays and these were therefore excluded from the analysis. A total of 51 out of 81 participants (63%, 95% CI: 52.1–72.7) were admitted to hospital for an inappropriate duration and 46 (90%) of these admissions were too long.

Prolonged LoS was frequently caused by a number of problems. Delays relating to the undertaking of investigations, planning for discharge (e.g. referral delays); treatment initiation; and failure to discharge the patient when medically fit were the most frequent causes of prolonged stays (see Table 2 for further details). In the cases where the patient was not discharged despite being medically fit there was no evidence of problems that may have caused a delay i.e. no evidence that there was a delay in the request/undertaking of investigations, referral requests etc.

**Table 2:** Causes of inappropriate lengths of stay

Cause of inappropriate length of stay	Frequency
Delayed undertaking/receipt of investigations	12
Delayed discharge planning	10
Delayed/inappropriate medical management	8
Not discharged when medically fit	8
Delayed provision of specialist medical/therapeutic services	8
Delayed referral to specialist medical/therapeutic services	6
Delayed transfer to rehab/sub acute facility	5
Further observation required	4
Delayed transfer to long term care	3
Delayed community social care provision	2
Suitable for outpatient care	2
Other	3
Total	71

Suggested actions to improve timely discharge included: a lead physician to coordinate patient care, increased frequency of senior medical review, improved coordination between medical and therapy services, improved planning (for example, identification of service and investigation requirements earlier in a patient's admission), prompt referrals to other services and completing investigations at an early stage in the admission were suggested. Also, faster access to the results of investigations, prompt provision of in-patient specialist health or therapeutic services and increased capacity of long term rehabilitation and sub-acute facilities would have facilitated the timely discharge of several participants.

### Factors associated with inappropriate admissions

Participants diagnosed with multiple sclerosis (MS), Parkinson's disease (PD) or dementia were most frequently admitted inappropriately (see Table 3 for further details). Participants with epilepsy were least frequently admitted inappropriately.

**Table 3:** Assessment of admission and LoS according to condition

	**Admission appropriate**		**LoS appropriate**		
**LTNC**	**Yes**	**No**	**Yes**	**No**	**Total**
	
	**% (n)**	**% (n)**	**% (n)**	**% (n)**	
	
Multiple sclerosis	56 (5)	44 (4)	0 (0)	100 (9)	100%
Parkinson's Disease	64 (7)	36 (4)	30 (3)	70 (7)	100%
Dementia	75 (5)	25 (3)	25 (2)	75 (6)	100%
Stroke	78 (31)	22 (9)	33 (15)	67 (25)	100%
Epilepsy	81 (13)	19 (3)	25 (5)	75 (10)	100%
Other	67 (6)	33 (3)	56 (5)	44 (4)	100%
					
Multiple LTNCs	74 (17)	26 (6)	8 (2)	92 (22)	100%

Participants who lived in their own home compared to a residential/nursing home were significantly more likely to be admitted inappropriately (unadjusted OR: 3.43, CI: 0.95–12.39, p = 0.036). No other variables were found to be significantly associated with an inappropriate admission (see Table 4). Participants who had been admitted to hospital via the Emergency Department and those who presented with a neurological problem were most frequently admitted inappropriately.

**Table 4:** Factors associated with inappropriate admissions and lengths of stay: Univariate analyses

		**Admission appropriateness**			**Length of stay appropriateness**		
		**Yes**	**No**	**P Value**	**Yes**	**No**	**P Value**
		
		**% (n)**	**% (n)**		**% (n)**	**% (n)**	
		
**Gender**	**Female**	70.7 (41)	29.3 (17)	0.678	37.5 (15)	62.5 (25)	0.932
	**Male**	74.1 (43)	25.9 (15)		36.6 (15)	63.4 (26)	
**Admitted via the emergency department?**	**Yes**	68.1 (47)	31.9 (22)	0.210	40.0 (18)	60.0 (27)	0.537
	**No**	78.7 (37)	21.3 (10)		33.3 (12)	66.7 (24)	
**Presented with a neurologically related problem?**	**Yes**	64.3 (27)	35.7 (15)	0.315	34.6 (9)	65.4 (17)	0.343
	**No**	73.7 (42)	26.3 (15)		46.3 (19)	53.7 (22)	
**Place of residence**	**Nursing/residential home**	88.0 (22)	12.0 (3)	0.049*	33.7 (7)	66.7 (14)	0.683
	**Own home**	68.1 (62)	31.9 (29)		38.3 (23)	61.7 (37)	
**Household members**	**Lives with others**	69.4 (43)	30.6 (19)	0.795	54.8 (23)	45.2 (19)	< 0.001*
	**Lives alone**	66.7 (20)	33.3 (10)		5.3 (1)	94.7 (18)	
**Receives home help**	**Yes**	74.3 (26)	25.7 (9)	0.262	25.0 (6)	75.0 (18)	0.058
	**No**	62.7 (32)	37.3 (19)		50.0 (16)	50.0 (16)	
**Referred to in-patient physiotherapy**	**Yes**	n/a	n/a	n/a	25.0 (10)	75.0 (30)	0.027*
	**No**				48.8 (20)	51.2 (21)	
**Referred to social services**	**Yes**	n/a	n/a	n/a	9.1 (1)	90.9 (10)	0.039*
	**No**				41.4 (29)	58.6 (41)	
**Transferred on discharge**	**Yes**	74.2 (66)	235.8 (23)	0.155	18.2 (2)	81.8 (9)	0.164
	**No**	57.0 (11)	42.1 (8)		40.0 (28)	60.0 (42)	

		**Median (IQR)**	**Median (IQR)**	**P Value**	**Median (IQR)**	**Median (IQR)**	**P Value**

**Age (119)**		71.5 (53.3–78.5)	74.0 (59.3–83.0)	0.177	72.5 (I62.5-81.0)	76.0 (58.0–83.0)	0.984
**Number of LTNCs (119)**		1 (1.0–2.0)	1 (1.0–2.0)	0.876	1 (1.0–2.0)	1 (1.0–1.0)	0.016*
**Number of presenting complaints (119)**		1 (1.0–2.0)	1 (1.0–2.0)	0.829	1 (1.0–1.0)	2 (1.0–2.0)	0.040*
**FIM score (73) (Score range 1–126, 126 indicates no impairment)**		111.0 (103.0–120.0)	113.0 (103.0–120.0)	0.873	103.0 (96.0–122.0)	107.0 (45.0–115.0)	0.228
**GDNS (walking) (74) (score range 1–5, 5 indicates no impairment)**		2.0 (1.5–4.0)	2.0 (0.0–4.0)	0.616	2.5 (0.0–4.0)	3.5 (2.0–4.0)	0.194
**GDNS (speech) (74) (score range 1–5, 5 indicates no impairment)**		0.5 (0.0–0.0)	0.0 (0.0–2.0)	0.122	0.0 (0.0-0.0)	0.0 (0.0–3.0)	0.078
**MMSE (49) (score range 1–30, 30 indicates no impairment)**		24.0 (23.0–28.5)	26.0 (24.0–28.0)	0.445	24.0 (18.5–26.5)	26.5 (23.0–29.0)	0.119
**Length of stay (119)**		8.0 (4.0–13.0)	4.0 (2.0–13.0)	0.063	4.0 (2.0–8.0)	10.0 (5.0–14.0)	< 0.001*

* Statistically significant

### Factors associated with inappropriate lengths of stay

Participants with MS, PD, epilepsy and dementia frequently experienced an inappropriate LoS (see Table 3). The number of LTNCs, number of presenting complaints, whether or not the participant lived alone in their own home, referral to in-patient physiotherapy and referral to social services were significantly associated with an inappropriate LoS (see Table 4 and Table 5). All variables significantly associated with an inappropriate LoS in Table 5 were entered into a single logistic regression model. The number of LTNCs, the number of presenting complaints and whether the participant lived alone or not were found to be significant predictors of an inappropriate length of stay (see Table 6).

**Table 5:** Unadjusted odds ratios for inappropriate length of stay

**Variable**	**Unadjusted OR**	**95% CI**	**P value**
Number LTNC	5.40	1.19–24.45	0.029
Number presenting complaints	2.669	1.29–5.54	0.008
Lives alone in own home	21.79	2.66–178.53	0.004
Referred to social services	7.07	0.86–58.34	0.069
Referred to physiotherapy	2.86	1.11–7.33	0.029

**Table 6:** Multivariate logistic regression models for inappropriate length of stay

	**Length of stay**		
	
**Variable**	**Adjusted OR**	**95% CI**	**P value**
Number LTNC	7.29	1.06–50.26	0.044
Number presenting complaints	7.55	2.05–27.81	0.002
Lives alone in own home	38.72	3.86–388.10	0.002

(n = 61, Hosmer-Lemeshow = 0.989)

## Discussion

The prevalence of inappropriate admissions amongst patients with LTNCs was comparable (i.e. approximately a third) to statistics obtained previously in studies of other adult or older adult populations [1,4,5]. The duration of LoS was found to be inappropriate in approximately three quarters of cases, a relatively high figure compared to most similar studies involving adult, older adult or stroke patients [5,6,21-23]. However, the assessment of appropriateness did not consider local availability of services. This may have led to higher numbers of admissions/lengths of stay being deemed inappropriate than assessments that take into account existing services and/or examine each individual in-patient day.

One risk factor for an inappropriate admission, living in your own home compared to a residential/nursing home, was identified and this may relate to the availability of support for those who live in long term care facilities (where care is available 24 hours a day) compared to those who live in their own home. For an inappropriate LoS, a number of risk factors were identified relating to the health status of the participant (number of LTNCs and number of presenting complaints) and the social circumstances of the participant (whether or not they lived alone in their own home). Although participants who required transfer were not at significantly greater risk, the majority who were transferred from hospital to a rehabilitation or long term care facility had an inappropriate LoS, indicating that ensuring the timely transfer of patients to these facilities can be a difficult task.

There are a number of limitations of this study. Firstly, the optimum sample size for the study was not achieved, therefore, the confidence intervals produced are less precise (8% compared to 5%). However, the logistic models have been checked for a goodness of fit using their residuals against fitted value plots, the factors included in the models were statistically significant; therefore, the analysis is valid. Failure to recruit the desired number of patients was primarily due to difficulties in recruiting patients when neither consent nor assent could be obtained. For such patients, ethical permission was given for a clinician involved in the patient's care to collect information from their health records. However, prior to this it was necessary to establish that neither a family member or friend was visiting and a minimum time period of three days was given to determine this. Patients were frequently discharged during this time. Also, whilst recruitment took place at two hospitals only one researcher was available to recruit patients. A large proportion of eligible patients were therefore discharged before the researcher could meet with the patient's family/friends to discuss the study. It is possible that such patients had greater levels of impairment (reflected in their inability to give informed consent) and would have been more dependent on services. The population recruited may be skewed towards those who were less physically and cognitively impaired, thus, the possibility of selection bias cannot be ruled out. Secondly, assessment of cognition, activities of daily living and neurological disability were only completed for those who were physically and mentally able to take part in an interview, or where a relative was able to give the information. The scores may not, therefore, reflect the LTNC population accurately. Thirdly, the number of inappropriate in-patient days was not examined due to time constraints. Lengths of stay that were deemed inappropriate therefore had a minimum of one day delay. Given the restricted capacity of acute beds and the potential risk of hospitalising patients inappropriately, it was felt that even a one day delay had great clinical significance. In order to justify rearrangement of services and examine cost savings, future research in this area would be required.

It is also noteworthy that in this study an expert panel method was used. Whilst this method best served the study aims, the limitations must be noted. In any group situation there may be psychosocial influences, for example those who are less confident in their answers may choose to conform with more confident group members [36]. Also, the professional backgrounds of the experts have been shown to have an impact on outcomes. For example, consultants have been shown to identify alternative forms of care less often than their counterparts in general practice [7]. In order to reduce the impact of psychosocial influences members were asked to consider the information in isolation initially and provide the researcher with a preliminary decision. This allowed the researcher to check whether any members consistently changed their decisions to agree with another. The panel included an acute care consultant, a rehabilitation consultant and a general practitioner. The tendency of one professional group to find more or less admissions/lengths of stay inappropriate would therefore have been balanced.

This study highlights several areas where interventions may reduce the incidence of inappropriate admissions and/or LoS. A large number of patients required assessment/observation in an ambulatory setting rather than an admission to hospital. It is important to ensure, therefore, that clinical decision unit models are equipped, designed and resourced to allow detailed assessments whilst preventing Emergency Departments and hospital wards from becoming congested. Also, for the participants studied, prompt access to specialist neurological advice would have been advantageous. Such advice may have prevented participants being admitted to wait for this advice, however, further research would be needed to confirm this. Also, this would help to meet the quality requirements laid out in the National Service Framework for Long Term Conditions, i.e. that people admitted with a neurological emergency should be assessed and treated in a timely manner by teams with the appropriate neurological skills (quality requirement three) and that specialist clinicians should be consulted when patients with LTNCs are admitted (quality requirement 11) [10]. It appears that access to some secondary care services needs to be more immediate, if unnecessary or extended hospital admissions are to be minimised. Increased availability of social care services, especially 'out of hours', has long been called for and our findings indicate that a number of inappropriate admissions documented in this study could have been avoided if such services had been accessed. Calls for increased social care services are not new and are an area of policy commitment, for example in the NHS plan, the NSF for older people and NSF for LTNCs [8,10,37]. Before revising services, or investing in new ones, it is essential that the barriers which impede access to existing services are examined fully and the relationships between agencies are understood. Internal reconfiguration of the way in-patient care is managed may help to ensure that patients are discharged when medically fit. Increased frequency of senior medical reviews and the allocation of a single lead physician for each case may tackle a number of problems. Junior physicians may not need to wait, for example, for advice from a senior colleague before a discharge is arranged and duplication of work may be avoided. However, the impact of any change on the remainder of the system would need to be examined. Before designing or reconfiguring interventions additional research would be required to assess the preferences and perceptions of service users and clinicians involved in the care of patients with LTNCs.

## Conclusion

A proportion of patients with LTNCs have been shown to be admitted to hospital when it was not medically necessary and to have remained in hospital for an inappropriate period. Patients with LTNCs may be at increased risk of developing hospital associated complications. In many cases ambulatory services or alternative community services exist which could have met the needs of participants and would have eliminated the need for admission. Increased access to ambulatory services such as clinical decision units and same day access to nursing home beds or emergency home care is needed. Identification of specific barriers which prevent services being accessed, from both the secondary and primary care perspective, to prevent inappropriate admission is required to determine where specific intervention is most needed. Organisational inefficiencies such as poor discharge planning and failure to recognise when patients are ready for discharge continue to exist. Increased frequency of senior medical review, early identification of requirements with timely referral to in-patient services and development of discharge plans are practical suggestions which could improve efficiency of discharge procedures.

## Competing interests

The authors declare that they have no competing interests.

## Authors' contributions

CLH designed the study, collected study data, performed the analysis and interpretation of data and produced the first draft of the manuscript. MFP provided assistance with the design of the study, provided support to CLH during the collection of data, and assisted with the revision of the manuscript. LLP provided assistance with the design of the study and assisted with the revision of the manuscript. BJP provided assistance with the design of the study, provided support to CLH during the collection of data, and assisted with the revision of the manuscript. AF provided assistance with the statistical design of the study, performed the sample size calculation, assisted with the analysis and interpretation of the data and assisted with the revision of the manuscript. All authors read and approved the final manuscript.

## Pre-publication history

The pre-publication history for this paper can be accessed here:


